# Supplementary material for: Sensitivity and Resistance of Oncogenic RAS-Driven Tumors to Dual MEK and ERK Inhibition
Source: Cancers (Basel). 2021 Apr 13;13(8):1852. doi: 10.3390/cancers13081852 (PMC8069437; doi:10.3390/cancers13081852)

Full unedited gel for Figure 1D

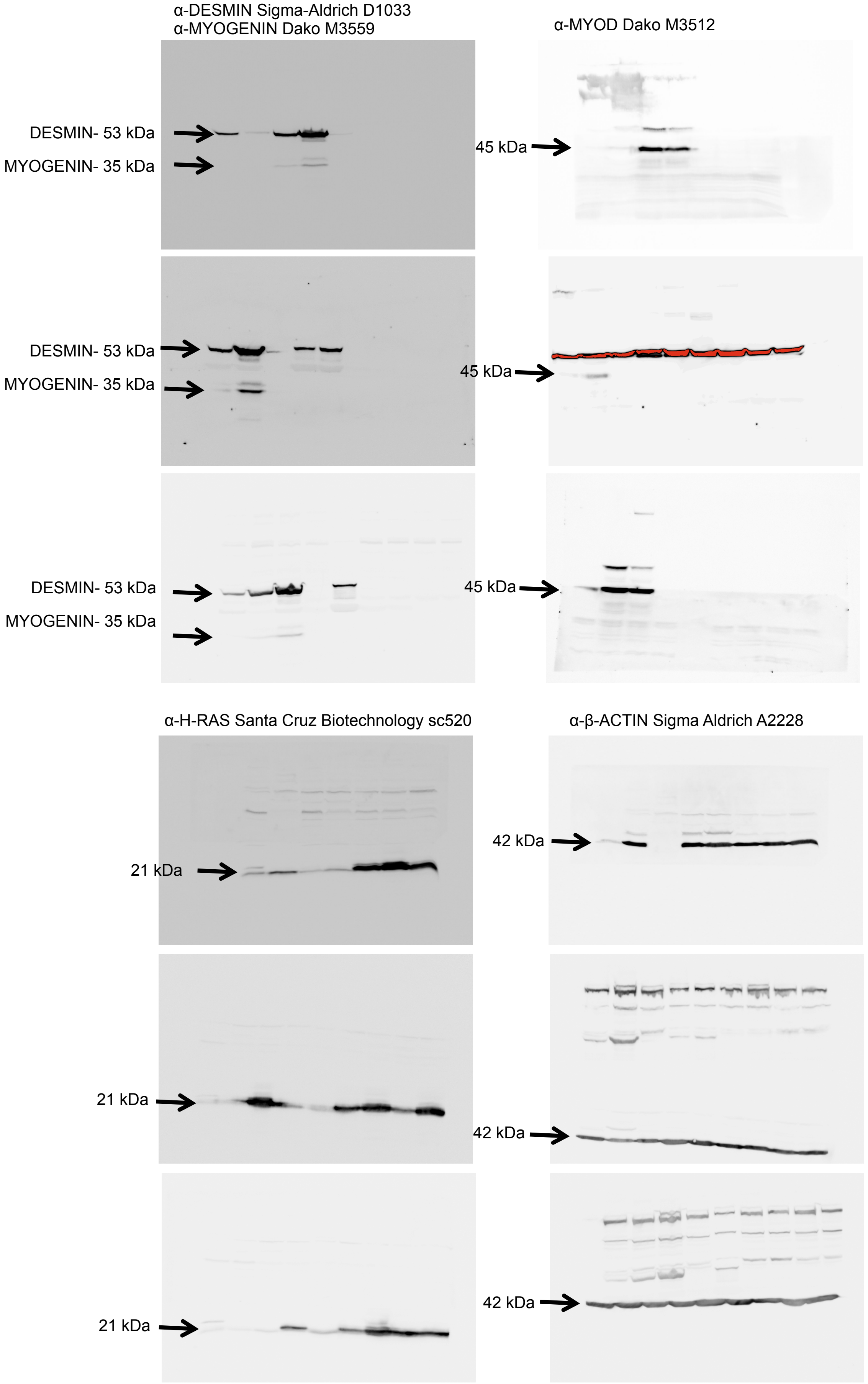

Full unedited gel for Figure 2E

α-phospho-Y1068-EGFR (D7A5)
Cell Signaling 3777

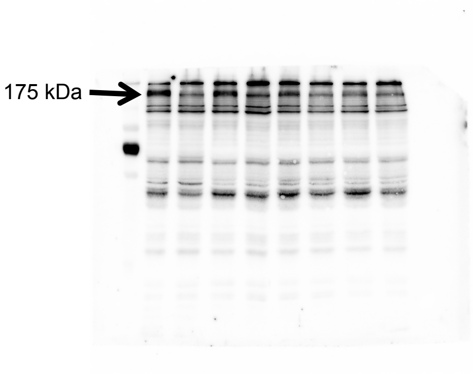

α-Total-EGFR (D38B1)
Cell Signaling 4267

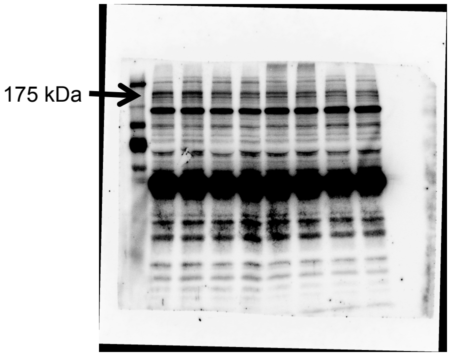

α-BRAF (H-145)
Santa Cruz Biotechnology, sc-9002

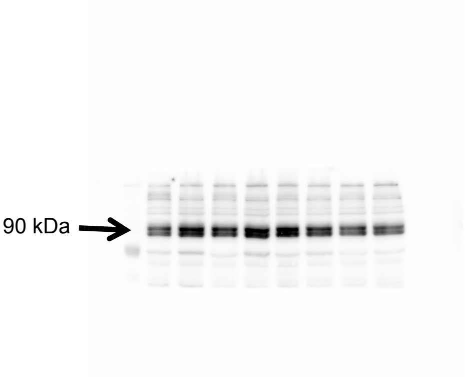

α-CRAF (C-12)
Santa Cruz Biotechnology, sc-133

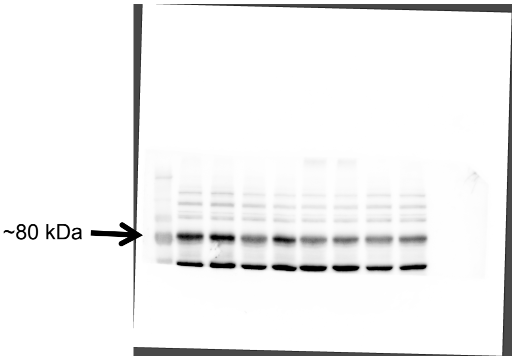

α-Total-MEK 1/2 Cell Signaling 9122

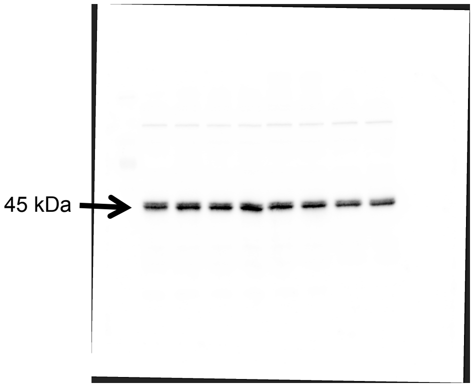

α-Phospho-S217/S221-MEK 1/2 (41G9)
Cell Signaling 9154

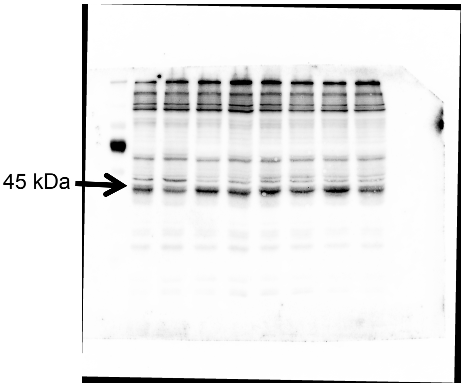

α-GAPDH Abcam, ab9484

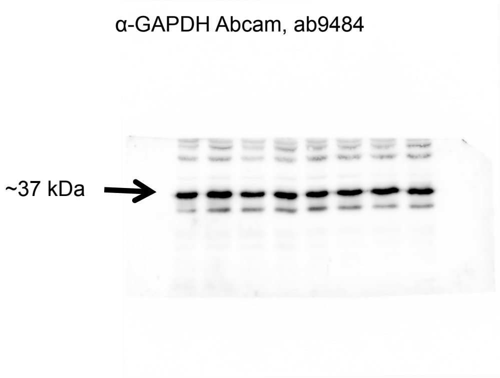

α-GAPDH Abcam, ab9484

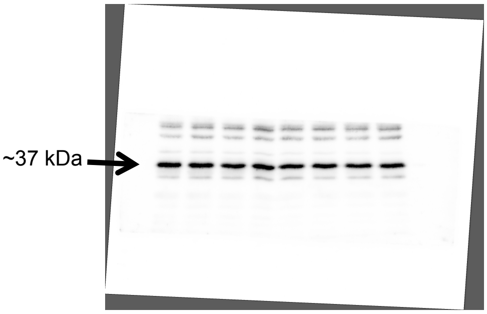

Full unedited gel for Figure 2F

α-Phospho-T308-AKT (C31E5E)
Cell Signaling 2965

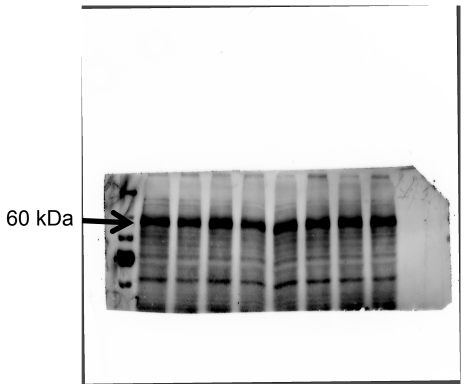

α-Total-AKT Cell Signaling, 9272

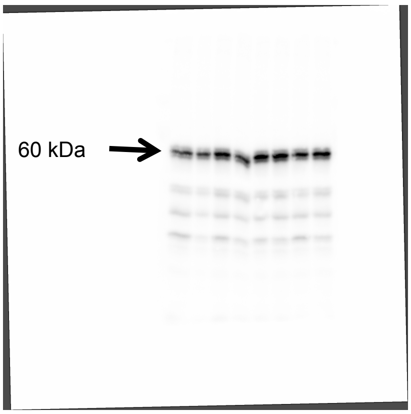

α-Total-ERK (137F5) Cell Signaling 4695S

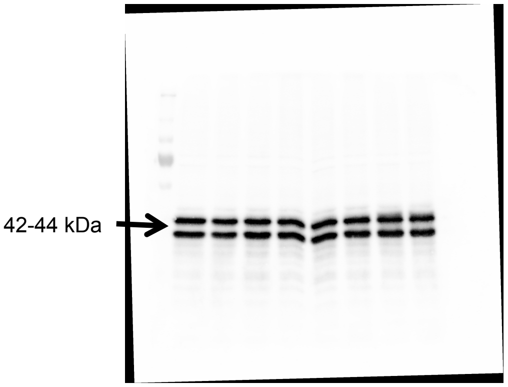

α-Phospho-T202/Y204-ERK Cell Signaling 9101

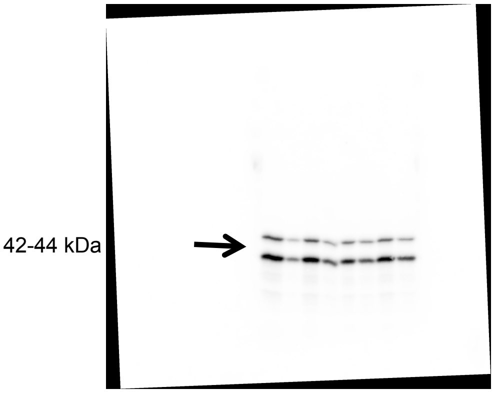

α-GAPDH Abcam, ab9484

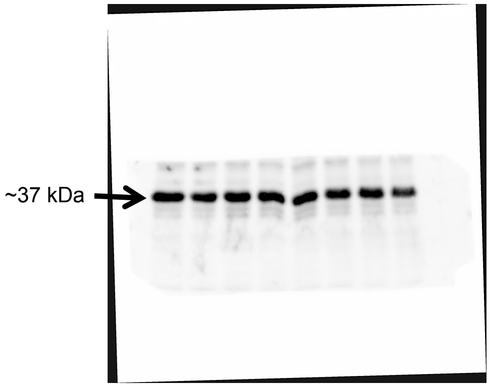

α-GAPDH Abcam, ab9484

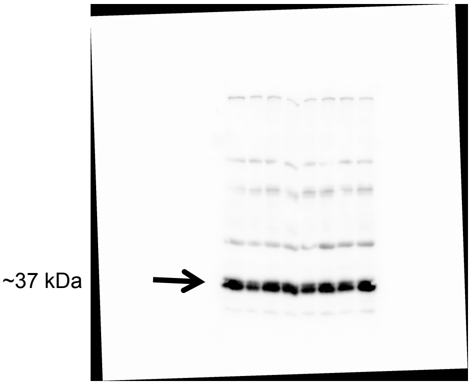

Full unedited gel for Supplemental Figure 6

α-VINCULIN (EPR8185) Abcam ab129002
α-Total-ERK (137F5) Cell Signaling 4695S

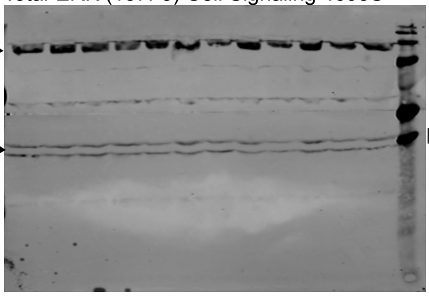

α-VINCULIN (EPR8185) Abcam ab129002
α-Phospho-T202/Y204-ERK Cell Signaling 9101

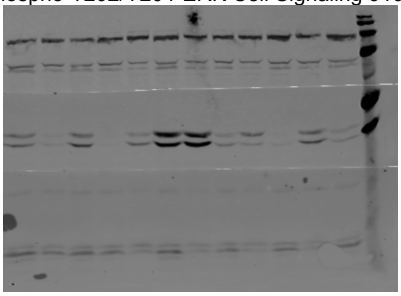

α-VINCULIN (EPR8185) Abcam ab129002

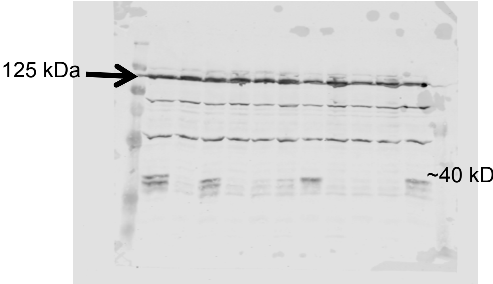

α-Phospho-S265-FRA1 (D22B1)
Cell Signaling 5841

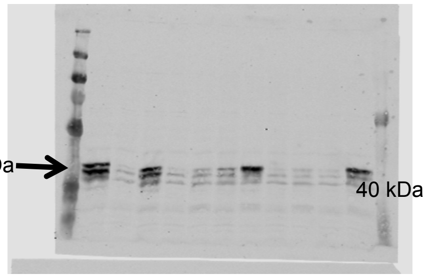

α-Total-FRA1 (D80B4) Cell Signaling 5281

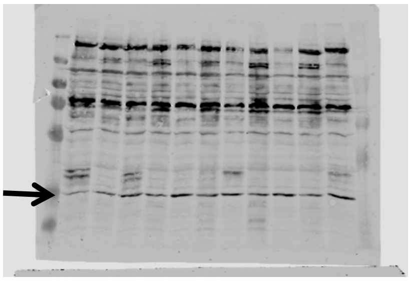

Supplement: Supplementary file 1 [file cancers-13-01852-s001.zip › cancers-1169220original.pdf]
