# Supplementary material for: Sensitivity and Resistance of Oncogenic RAS-Driven Tumors to Dual MEK and ERK Inhibition
Source: Cancers (Basel). 2021 Apr 13;13(8):1852. doi: 10.3390/cancers13081852 (PMC8069437; doi:10.3390/cancers13081852)
Supplement: Supplementary file 1 [file cancers-13-01852-s001.zip › cancers-1169220supple.pdf]

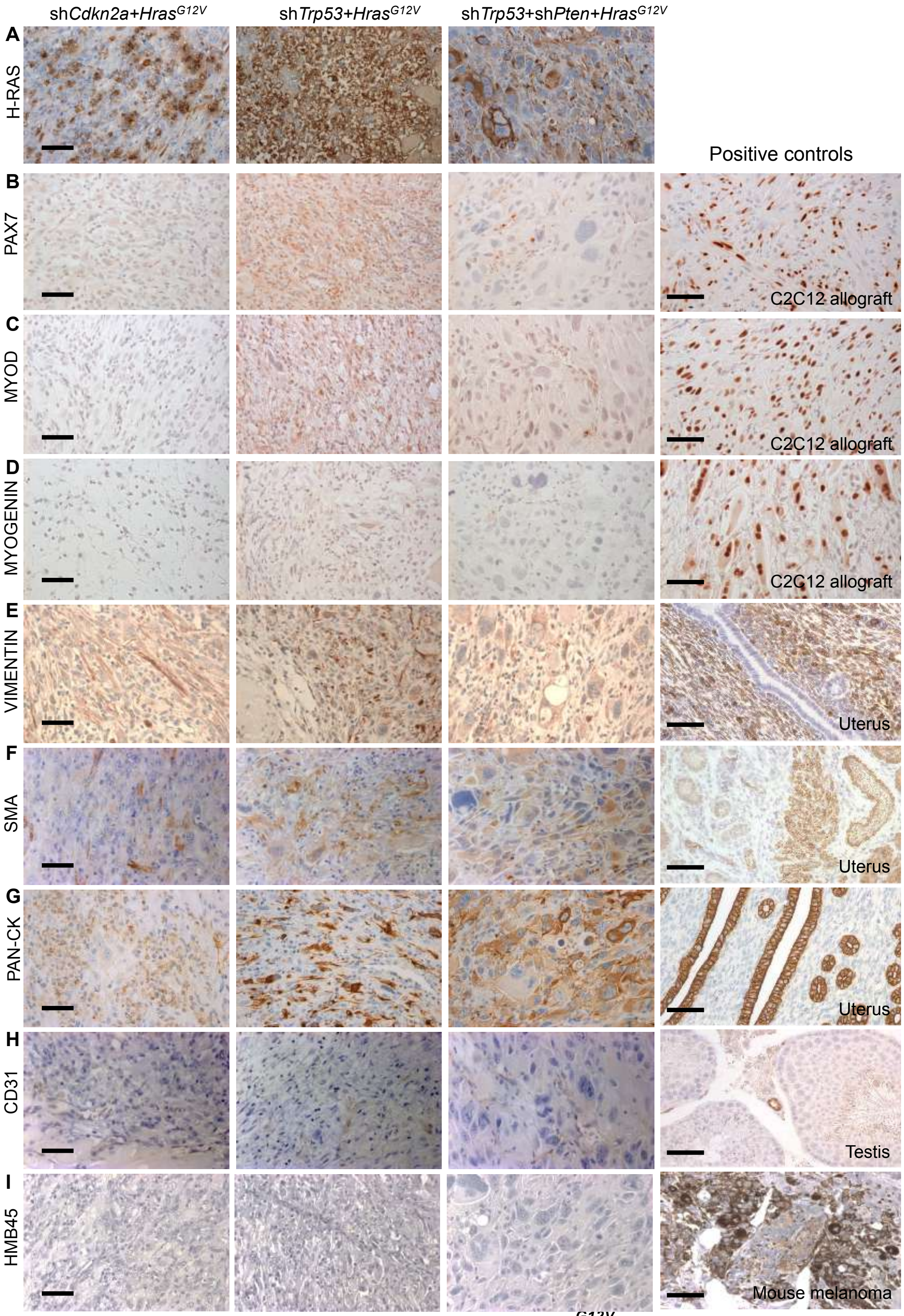

Figure S1. Intramuscular injection of oncogenic *Hras*^{G12V} with knockdown of *Trp53*, *Pten* or *Cdkn2a* causes non-myogenic undifferentiated pleomorphic sarcoma in mice. Immunohistochemical stainings of tumours arising from the intramuscular injection of MuLE viruses expressing either shRNA-*Cdkn2a* + *Hras*^{G12V}, shRNA-*Trp53* + *Hras*^{G12V} or shRNA-*Trp53* + shRNA-*Pten* + *Hras*^{G12V} in SCID/Beige mice. Positive controls for each antibody include C2C12 allograft tumours, the indicated mouse tissues or a mouse melanoma. PAN-CK = pan-CYTOKERATIN, SMA = Smooth muscle actin. Scale bars = 50µm. Stainings are representative of 7, 12 and 2 independent tumours of each genotype respectively.

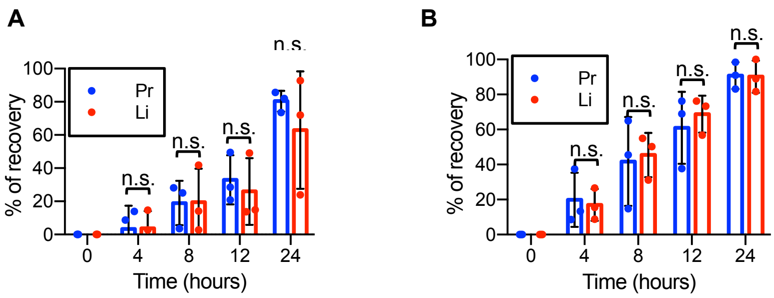

Figure S2. (Additional information related to Figure 1) Time course of recovery of wounds induced by scratch assay on confluent cells. Experiments performed on UPS 1 Pr and UPS 1 Li (**A**) and on UPS 9 Pr and UPS 9 Li (**B**) complementary to experiments performed on UPS 7 Pr and UPS 7 Li and showed in Figure 1E. Mean ± std. dev. are shown (n=3). In all matching pairs of cells the differences between primary tumour and metastatic cell lines were not statistically significant ($p>0.05$, unpaired t-test Holm-Sidak method).

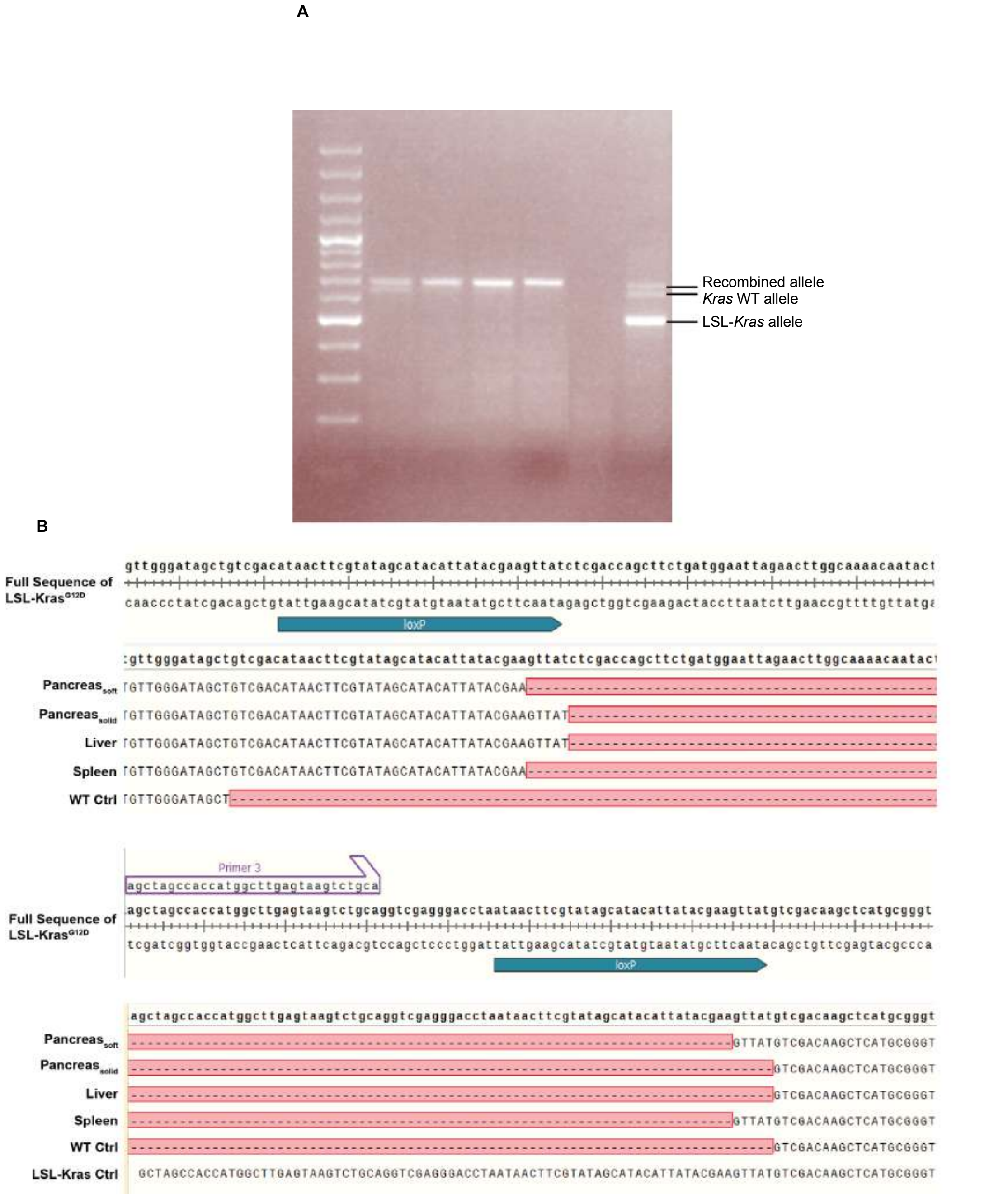

Figure S3. *Kras* recombination status in isolated KPC cell lines.

A. Representative results of *Kras* recombination-specific PCR yielding bands of 500 bp (LSL), 622 bp (WT) and 650 bp (1 *lox*P, recombined).

B. Sequencing results of the isolated bands. Red bars indicate sequence mismatch. Note that WT control does not contain *lox*P sites, while in recombined cells only one *lox*P site is left after recombination.

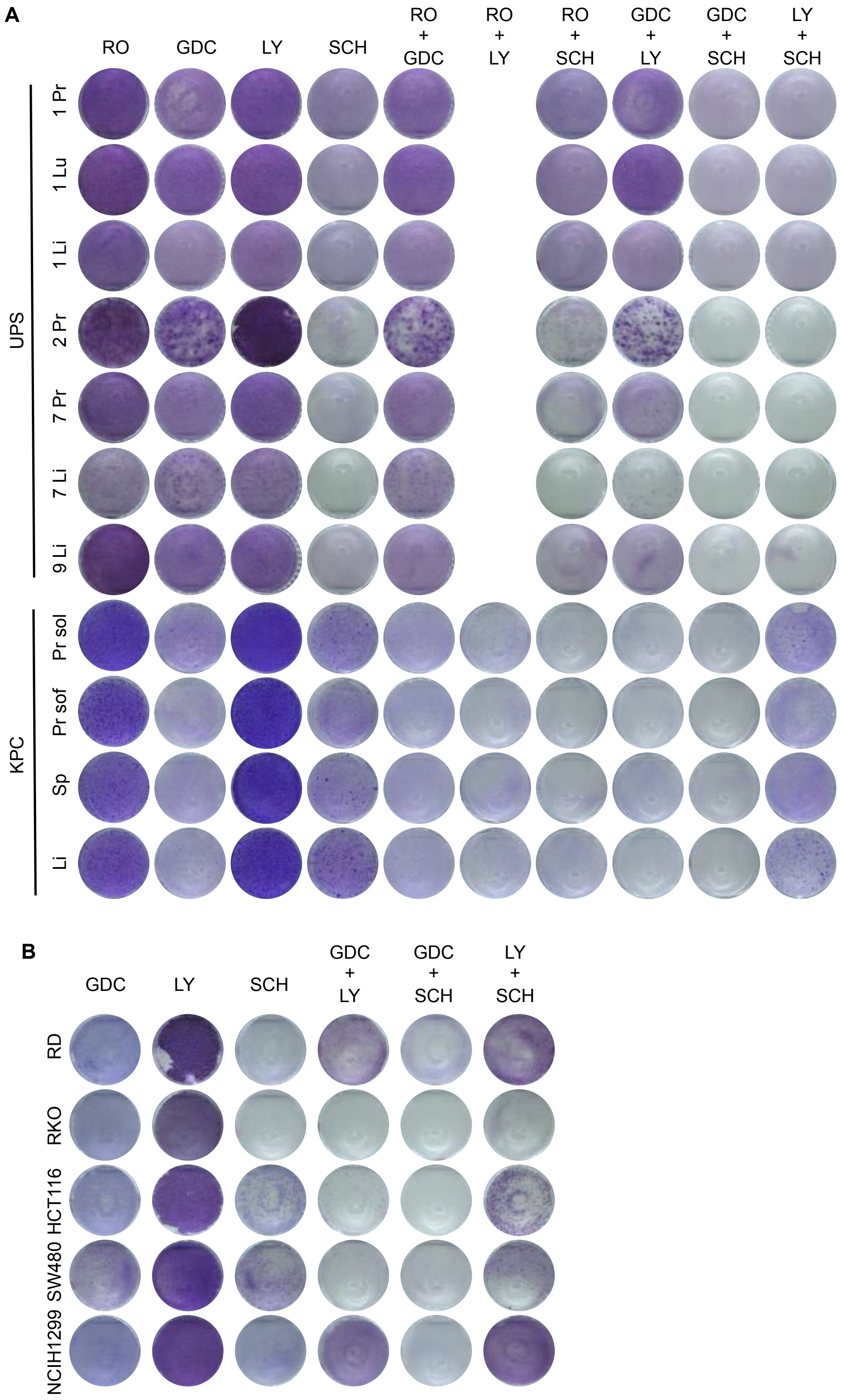

Figure S4. Dual MEKi plus ERKi prevents the emergence of drug resistance.
A-B. Images of UPS and KPC cell lines (A) as well as human tumour cell lines (**B**) cultivated for 14 days with the indicated inhibitors (1μM) and stained with Crystal Violet solution. Treatment of RO+LY was not performed on UPS cell lines.

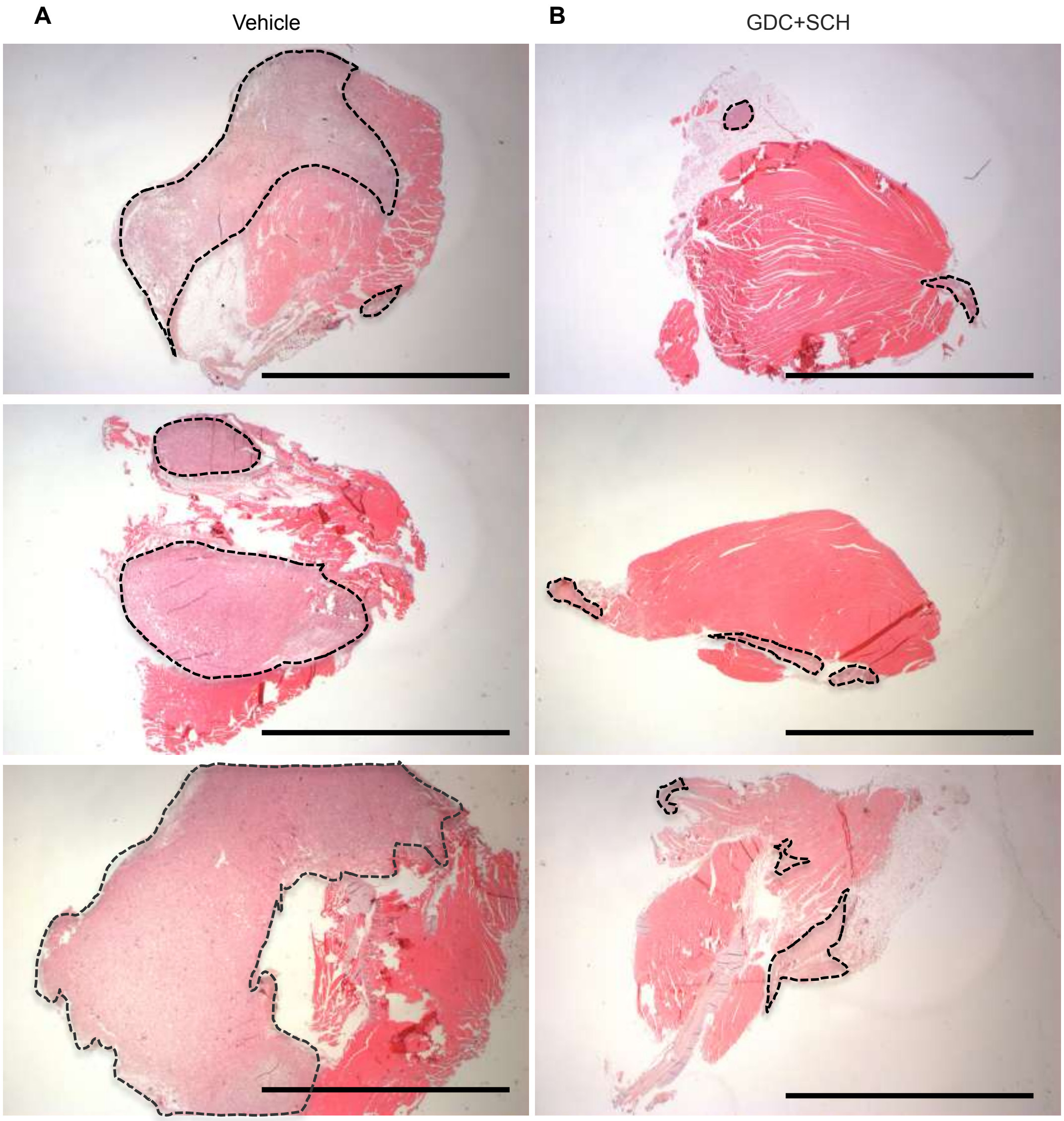

Figure S5. (Additional information related to Figure 5).
H&E stainings of (low-magnification pictures of) tumours developing in mice treated with vehicle (**A**) and tumour nests in mice treated with GDC+SCH (**B**). ROIs define the tumour area within the muscular tissue. Scale bar= 5mm

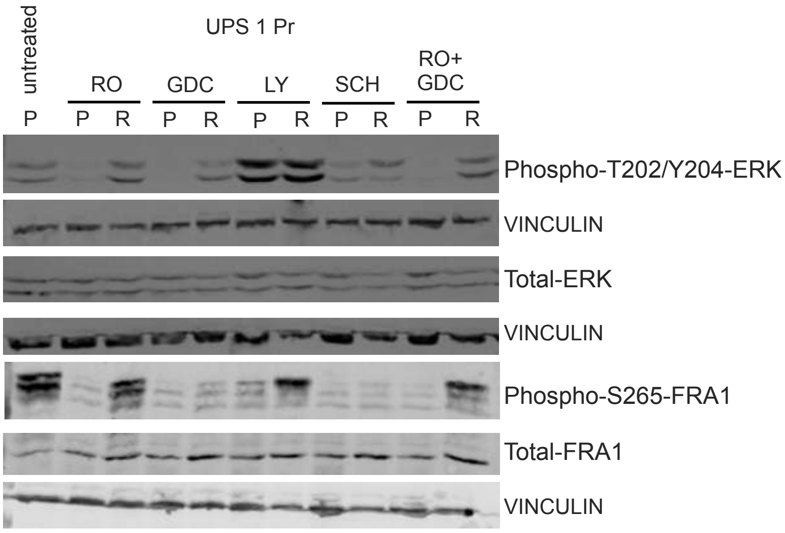

Figure S6. Analysis of signalling downstream of RAS in drug resistant cells. Western-blot performed on UPS 1 Pr parental (P) and resistant (R) cell lines to each of the indicated drugs. Each set of P and R cells were treated for 24 hours with the indicated respective inhibitor (1μM).

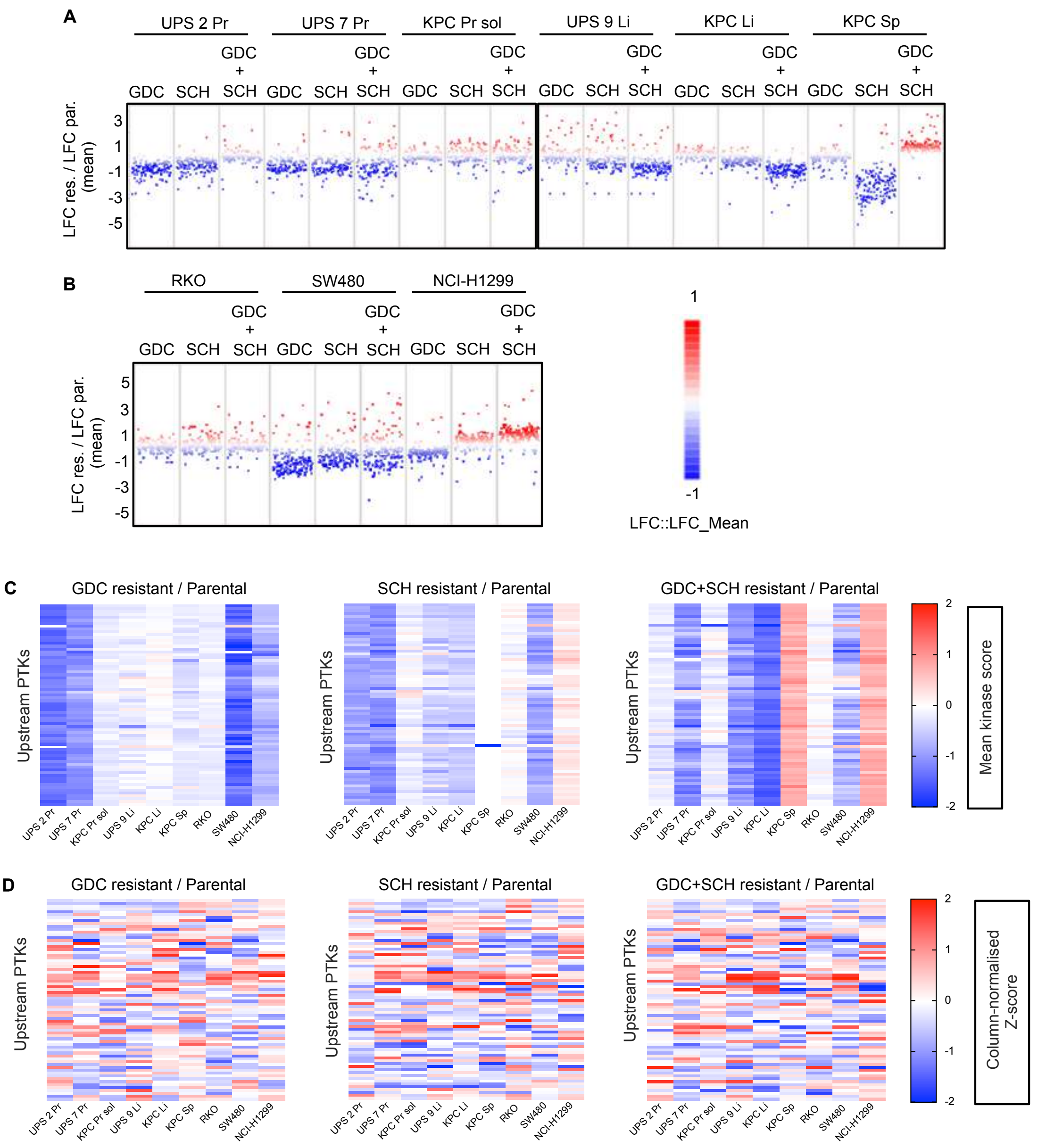

Figure S7. Patterns of PTK activity in resistant cells.

A-B. Log$_2$ Fold Change (LFC) distribution of upstream PTK kinase activity scores in resistant cell lines relative to their parental untreated counterpart in mouse (**a**) and human (**b**) cell lines.

C. Heatmaps depicting ratios of upstream kinase activity scores of resistant relative to parental cells organised by kinases on the y-axis.

D. Heatmaps of column-normalised Z-scores based on upstream kinase activity scores of resistant relative to parental cells organised by kinases on the y-axis. The Z-score acts as an intra-sample normalisation to highlight relative differences in the activity of individual kinases compared to all kinases within the sample.

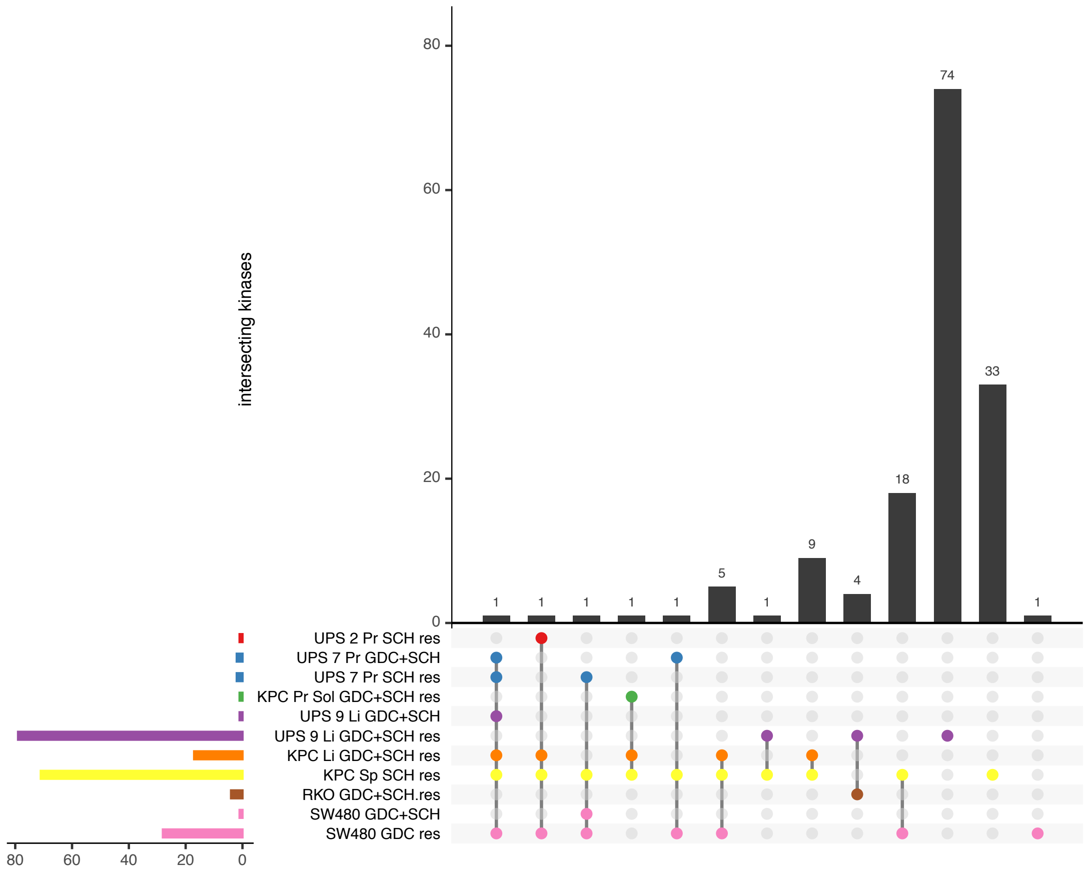

Figure S8. Overlapping donwregulated kinases

UpSet plot of the overlapping downregulated kinases with LFC mean kinase score less than or equal to -1.5. The bar plot (top) shows the number of intersecting downregulated kinases among the sets of cell lines that are indicated in the matrix below. The bar plots on the left show the total number of downregulated kinases in each cell line.
